# Hemophagocytic Syndrome Associated with Immune Reconstitution Inflammatory Syndrome in a Patient with AIDS Related Burkitt's Leukemia/Lymphoma

**DOI:** 10.1155/2014/308081

**Published:** 2014-05-27

**Authors:** Metin Kanıtez, Mahir Kapmaz, Nilufer Alpay, Fatih Selcukbiricik, Atahan Çağatay, Reyhan Diz-Küçükkaya

**Affiliations:** ^1^Department of Medical Oncology, Sisli Education and Research Hospital, Istanbul, Turkey; ^2^Department of Clinical Microbiology and Infectious Disease, Istanbul School of Medicine, Turkey; ^3^Department of Internal Medicine, Istanbul School of Medicine, Turkey; ^4^Department of Hematology, Istanbul Bilim University, Turkey

## Abstract

Highly active antiretroviral therapy (HAART) has markedly decreased human immunodeficiency virus- (HIV-) related mortality and the incidence of opportunistic infections. The dramatic reduction in HIV-1 RNA and increase in CD4 lymphocyte count mean a recovery in immune function. This restoration in immune function may be associated with paradoxical deterioration in subclinical opportunistic infections in some patients, a condition called immune reconstitution inflammatory syndrome (IRIS). IRIS, a “paradoxical” inflammatory response to either previously treated or subclinical infections or noninfectious diseases, can manifest during the restoration phase of immunity hemophagocytic syndrome (HS) which is a very rare complication in patients with acquired immune deficiency syndrome (AIDS). We describe a case of hemophagocytic syndrome associated with IRIS in a patient with AIDS related Burkitt's leukemia/lymphoma (BL). IRIS was probably the cause of hemophagocytosis for our patient. Zoster infection may facilitate to IRIS. With the increasing number of people with HIV infection and the accompanying use of HAART, much more clinical manifestations of IRIS will be experienced especially in patients given high dose chemotherapy, just like in our case.

## 1. Introduction

Highly active antiretroviral therapy (HAART) has markedly decreased human immunodeficiency virus- (HIV-) related mortality and the incidence of opportunistic infections. The dramatic reduction in HIV-1 RNA and increase in CD4 lymphocyte count mean a recovery in immune function. This restoration in immune function may be associated with paradoxical deterioration in subclinical opportunistic infections in some patients, a condition called immune reconstitution inflammatory syndrome (IRIS) [1, 2]. IRIS may have very different clinical manifestations [1]. Hemophagocytic syndrome (HS) is a very rare complication in patients with acquired immune deficiency syndrome (AIDS), with a pathogenesis of unclear certainty [3]. Here, we describe a case of hemophagocytic syndrome associated with IRIS in a patient with AIDS related Burkitt's leukemia/lymphoma (BL).

## 2. Case

A 39-year-old woman had a diagnosis of BL with an Ann Arbor stage IVB in June 2008. She had thoracic and intra-abdominal lymphadenopathies with bone marrow involvement. HIV screening was positive with a CD4 lymphocyte count of 274 cells/mL and HIV-1 RNA of 124.000 copies/mL. The serology of* Toxoplasma gondii*, cytomegalovirus, herpesvirus, and hepatitis A, B, and C viruses was negative. Both hyper-CVAD (hyperfractionated cyclophosphamide, vincristine, doxorubicin, and dexamethasone) for BL and an antiretroviral therapy consisting of zidovudine-lamivudine with ritonavir-lopinavir were initiated.

**Figure 1:**
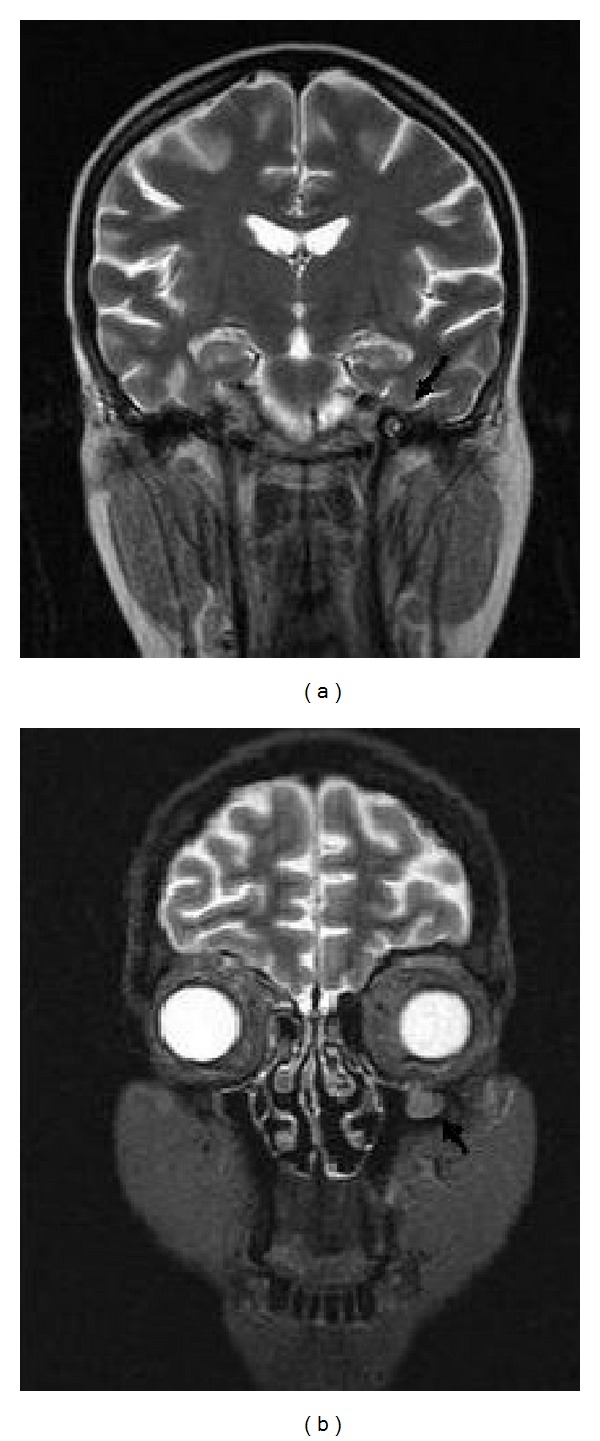
The cranial and orbital sagittal T2-weighted magnetic resonance image revealing a thickening of the third division of trigeminal nerve (a) and a soft tissue development of one centimeter diameter in the roof of left maxillary sinus (b).

**Figure 2:**
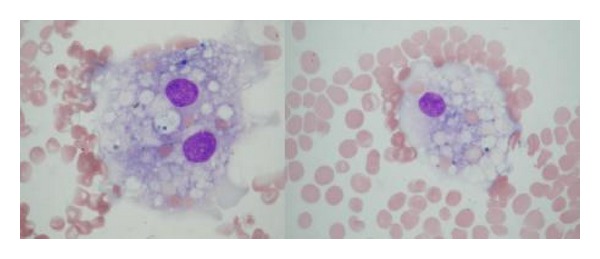
The bone marrow aspiration revealing active hemophagocytosis with prominent mature histiocyte phagocytosis of red blood cells (May-Giemsa stain, ×100).

In October 2008, a complete remission of the BL was shown by bone marrow biopsy and radiological findings, with an increasing count of CD4 lymphocytes to 412 cells/mL and HIV-1 RNA of 135 copies/mL. Then, ptosis developed on her right eye, with a facial palsy on her left. Cranial and orbital magnetic resonance images revealed a thickening of the third division (V3) of trigeminal nerve and a soft tissue development in the roof of left maxillary sinus (Figures 1(a) and 1(b)). The CSF analysis was normal with microbiological screening for* Cryptococcus neoformans*, cytomegalovirus (CMV), herpesvirus (HSV), and* Mycobacterium* found to be negative, and no malignant cell was observed. After two days, zosteriform lesions appeared on her right side reflecting V1 of trigeminal nerve. With a ten-day treatment of acyclovir, the lesions resolved. However, ptosis and facial palsy persisted with findings of painless ulcerative gingivitis, subfebrile fever, and hepatosplenomegaly. Blood and urine cultures were negative for bacteria, mycobacteria, and fungi. Laboratory tests showed pancytopenia (hemoglobin, 7 g/L; white blood cells, 1100 mL; platelets, 40.000 mL). Prothrombin and activated partial thromboplastin time were normal and lactate dehydrogenase was elevated (768 IU/L). The liver enzymes, vitamin B12, and folic acid were in normal range. The bone marrow aspiration revealed a hypocellularity with no malignant cells. Besides this, active hemophagocytosis with prominent phagocytosis of erythroid precursors was detected (Figure 2). Both Parvovirus IgM and Epstein Barr VCA IgM were negative. PCR for HSV types I and II and CMV were found to be negative. The repeated serology for* Toxoplasma* and* Cryptococcus neoformans* antigen were negative in serum. At this point, a diagnosis of IRIS was considered. The antiretroviral therapy was stopped. Although methyl prednisolone (1 g i.v. daily for three days) followed by intravenous immunoglobulin (1000 mg/kg daily for two days) was given, the patient died with a multiorgan failure during followup.

## 3. Discussion

IRIS, a “paradoxical” inflammatory response to either previously treated or subclinical infections or noninfectious diseases, can manifest during the restoration phase of immunity [4]. Its diagnosis is clinical and requires excluding alternative conditions. IRIS may give rise to a heterogeneous range of clinical presentations [1]. Otolaryngological [2, 5] and neurological manifestations [6, 7] are also reported. With this regard, the nonspecific soft tissue developments in maxillary sinus and facial palsy in our patient are thought to be associated with IRIS.

Besides this, we diagnosed a hemophagocytic syndrome in our case, which may be an interesting manifestation of IRIS. In the literature, similarly, HS is reported as a probable presentation of IRIS [8]. HS is a rare disorder with a similar pathophysiology of IRIS and characterized by overproliferation of mature histiocytes, hemophagocytosis, and upswing of inflammatory cytokines [9]. The decrease in cytotoxicity of natural killer cells and cytotoxic T cells with upregulation of *γ*-interferon probably plays a role in the pathogenesis of HS, leading to an increase in macrophage activation and plentiful secretion of proinflammatory cytokines [4]. Infectious agents, mostly viruses of herpes family, usually are the triggers. Malignant lymphomas, especially in adults, may be associated with HS. An autopsy study of 56 patients with AIDS diagnosed an approximate 20% rate of hemophagocytosis [3]. Recent reports have suggested that HS may be a manifestation of acute HIV infection [10]. Because no evidence of Burkitt's lymphoma was found at the time of HS in our patient, it was excluded as a trigger. The herpes zoster infection, however, might be associated with IRIS and HS in this case. Although the chemotherapy given to the patient for the lymphoma was not thought to have a role with HS or IRIS, it might delay the possible symptoms of IRIS in such a way preventing the recovery of CD4 counts.

The guidelines for treatment of IRIS are not well defined. Nonsteroidal anti-inflammatory drugs and systemic corticosteroids may be used for complications secondary to the exaggerated inflammatory process in IRIS [11]. In severe cases, HAART may be helpful to be stopped until the inflammatory condition and/or the infection taken under control [1, 4]. The treatment options of HS are corticosteroids, immunoglobulins, etoposide, antithymocyte globulin, cyclosporine, and stem cell transplantation [9]. We preferred to stop antiretroviral therapy and initiated high dose prednisolone together with i.v. immunoglobulin, in a useless attempt for the patient at the end.

In conclusion, IRIS was probably the cause of hemophagocytosis for this patient. Zoster infection may facilitate IRIS. With the increasing number of people with HIV infection and the accompanying use of HAART, much more clinical manifestations of IRIS will be experienced especially in patients given high dose chemotherapy, just like in our case.
